# Migraine – more than a headache: Women's experiences of living with migraine

**DOI:** 10.3109/09638288.2011.607211

**Published:** 2011-10-10

**Authors:** Stina Rutberg, Kerstin Öhrling

**Affiliations:** Department of Health Science, Luleå University of Technology, Luleå, Sweden

**Keywords:** Migraine, pain, qualitative research, women

## Abstract

**Purpose:**

In this qualitative study the aim was to explore the meaning of living with migraine.

**Methods:**

In-depth interviews were conducted with ten women about their experience of living with migraine. Halfway through the interview, the women drew a picture of what living with migraine is like, and the interview continued with the conversation being guided by the picture. The interviews were analyzed using a hermeneutic phenomenological method inspired by van Manen.

**Results:**

The analysis revealed an essence “Being obliged to endure a life accompanied by an unpredictable and invisible disorder" and three themes “Being besieged by an attack” “Struggling in a life characterized by uncertainty"and “Living with an invisible disorder."

**Conclusions:**

Migraine is a debilitating disorder which accompanies life in the sense that it or the threat of its return is always present, and yet invisible to others. The struggle of enduring life with migraine is worsened by the feeling of having an invisible disorder and of being doubted. There is a need to increase the knowledge among healthcare professionals about what it means to live with migraine, something this qualitative study offers.

## Introduction

Migraine is a common disorder that affects three times more women than men [1]. This disorder is characterized by a cycle of painful headaches with associated symptoms such as nausea, photophobia or phonophobia separated by apparently symptom-free periods [2]. However, the effects of migraine are not limited to the periods of pain associated with an attack as, in the periods between one attack and the next, migraine sufferers might experience fear and anxiety in anticipation of the next attack [3]. Thus, migraine can be viewed as an ongoing cycle of suffering, because it involves treating the current attack and worrying about the next one [4].

In 2001, The World Health Organization (WHO) listed migraine as being among the top twenty causes of disability [5]. Nevertheless, migraine is an often unrecognized and undertreated disorder [6]. Only about 50% of people fulfilling the criteria for migraine had received an appropriate diagnosis and treatment [7,8]. In addition, in a nation-wide survey in Sweden only one in four of the persons diagnosed with migraine had consulted a healthcare expert and 60% of those who had were not satisfied with the information or treatment that they had been offered in the consultations [9]. Brandes [10] suggests that physicians who understand the impact of migraine on their patients’ lives are better equipped to assist with managing migraines and to help their patients to regain control of their lives. Life with migraine is a burden as it is associated with high levels of headache-related disability [11] which is affected by the worry about future attacks and a lack of control over the illness [10]. This state of uncertainty impacts upon the ability to make plans and to engage in activities [12,13]. Furthermore, the burden of being afflicted with migraine extends to the family, social relationships and work, which also affects the quality of life [9,13-15].

**Implications for Rehabilitation**

- The meaning of living with migraine is experienced as having a debilitating disorder which accompanies life in the sense that it or the threat of its return is always present, and yet invisible to others.
- There is a need for healthcare professionals to increase their awareness of the meaning of living with migraine, to enable them to meet the needs of each person with migraine.

Persons with migraine manage their illness in a highly individual way and predominantly, use the traditional medical system, generally taking the form of prescription drugs [16]. The way they choose to manage their migraine was impacted by their own perceptions of their disability and even though those afflicted with migraine perceived it to be a serious problem, they viewed their illness as a less serious health issue than some others have to face [17]. Peters et al. [18] have elaborated on the process of the decision-making migraine sufferers go through, a process which developed over time and operated on a justification and consequence system. With the aim of exploring the concept of vigilance, Meyer [19] describes how, in their attempts to manage life with migraine, women were always prepared to prevent and to attempt to abort attacks. Although gaining a perspective of the management of the illness is important, it only gives us an insight into one aspect of what it means to live with migraine.

Two focus group studies [20,21] found that migraine has a negative affect on the quality of life of the person afflicted and on the person's family life, work and relationships. Furthermore, Ruiz de Velasco [20] pointed out that the psychological wellbeing of the migraine sufferer is frequently affected. Moloney et al. [22] concluded that, in midlife, women experienced migraine as a continuing presence in their life and that having migraine affected their relationships and their ability to manage their responsibilities. This knowledge give an insight into how migraine can influence different aspects of life, but, however, questions still remain to be answered about what it really means to be a person living with migraine. Both Peters et al. [17] and Moloney [22] state that more qualitative research is needed about the experience of migraine to help healthcare providers develop a deeper understanding of the experience their patients are undergoing. Thus, with the intention of increasing the understanding of migraine, the aim of this study was to explore the meaning of living with migraine.

## Methods

### Design

Phenomenological research is characterized by beginning in the life world and, according, to Dowling [23] it tries to distinguish the nature or essence of a phenomenon to better understand what the particular experience is like. This corresponded well with the aim of our study. A hermeneutic phenomenological method, as described by van Manen [24], was chosen to explore the meaning of living with migraine. To gain understanding of the meaning of a phenomenon it is necessary to reflect on the lived experience and, as van Manen ([24] p. 32) declared, “A true reflection on lived experience is a thoughtful, reflective grasping of what it is that renders this or that particular experience its special significance.” According to van Manen [24], the term essence is a description of a phenomenon, where the structure of lived experience is revealed in such a way that it is possible to grasp the nature and significance of the experience in a hitherto unseen way. Phenomenology also involves balancing the research context by considering the parts and the whole. Van Manen [24] argues that the ideas behind hermeneutic phenomenology acknowledge the interpretation of lived experience and believes that it is not possible to suspend or bracket pre-understandings or assumptions; rather they must be made explicit and therefore we made an effort to keep an open mind about the phenomenon and towards the participants during the study.

### Participants

The Swedish Migraine Association agreed to forward information about the study to members living in county of Norrbotten, Sweden. To be included in the study the participants had to be at least 18 years old, diagnosed with migraine and speak Swedish. Letters describing the purpose of the study were sent to all members (24 persons), and a prepaid envelope was included to be used for the reply. Eleven women and one man answered, saying that they would be interested in participating in the study. Two of those people were excluded from the study; one had recently moved to the south of Sweden and one had not been diagnosed as having migraine.

Ten women participated in the study; they were aged between 37 and 69 years old. They subjectively estimated the impact migraine had on their life using one of three grades, slight, medium or severe; of the 10 women, 4 classified the impact as medium and 6 classified as it as being severe. For eight participants, the migraine had either started in their late teens or in their early twenties. For two of the women, the migraine had started when they began menopause. The number of migraine attacks varied, 1-2 attacks per year for two women, 1-4 attacks per month for 6 women and 10-20 attacks per month for two women. Four women were working full-time, four part-time and, two had retired. Eight women lived with their husbands and two women maintained separate homes from their partners. Four women had children living at home and five had adult children who no longer lived at home.

### Data collection

The persons interested in participating were contacted by phone and additional information about the study was given. They all gave their written informed consent to participate in the study and the time and place of the interview was decided. Seven interviews took place at the home of the participant, and three interviews were conducted at the university, all in an undisturbed room. The interviews were performed by the first author. To capture the participant's experience of living with migraine, the interview started with the request: “Please tell me about your experience of living with migraine.” The interview was supported by questions like “How did you feel then?” and “Can you tell me more about…?"

**Figure 1:**
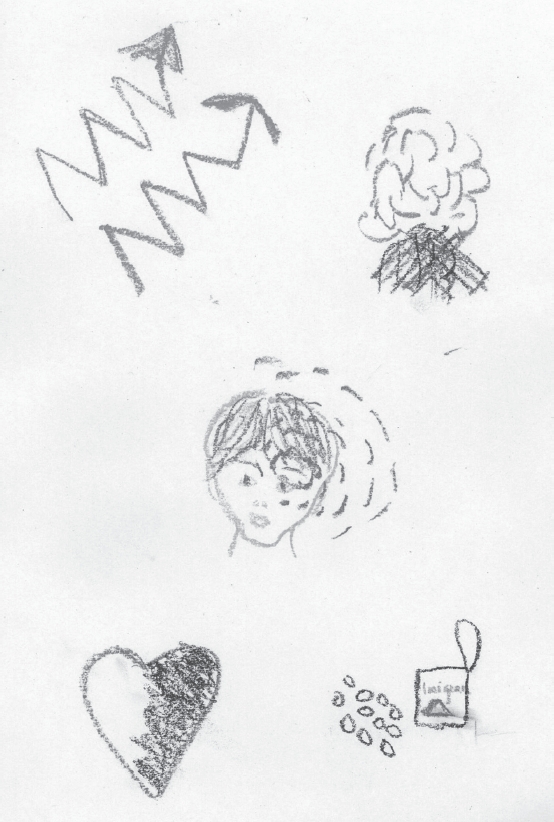
An example of a picture drawn during the interview by one participant.

An interview is limited by the participant's openness, perceptiveness and ability to recall the memory of the situation, in addition to the ability to verbalize his or her experience [25]. As pictures can be seen as a language of a nonverbal kind [24] and as Leitch [26] claims that pictures can give knowledge beyond the limitation of language, we chose to use drawings to facilitate the interview. When the interview reached the point where the participant did not have any more information to add to the narrative, the woman was asked to draw a picture of what it is like having to live with migraine (Figure 1). Thereafter the interview continued with narrations about the picture starting with the questions: “what is in the picture?” and “what feelings and associations do you get when you look at the picture?” Two of the participants chose not to draw a picture, one of them talked instead about a film to illustrate the picture she would have drawn. The interviews lasted for an average of two and a half hours, were tape-recorded and transcribed verbatim with indications of nonverbal pauses, sighs, gesticulation, laughs and weeping.

### Data analysis

The analyzing procedure, followed in this research, inspired by van Manen [24] involved interrelated phases; seeking meaning, theme analysis and interpretation with reflection, that considers the parts and the whole in the text. The search for meaning started with a holistic reading, which consisted of repeatedly listening, reading and re-reading the recorded and transcribed data to capture the fundamental meaning of living with migraine. Once complete, we formulated phrases that expressed the meaning. In the selective reading, the entire text was reread and phrases and sentence clusters that seemed to be thematic were marked as meaning units. As part of this selective approach, we asked: What does this unit reveal (the-matically) about the phenomenon? In the detailed reading, in contrast, the preliminary themes constructed during the selective reading were controlled by asking: “What does this really reveal about the nature of living with migraine?” to add nuance and deepen the understanding of the themes. Meaning units were considered and the themes were reread, combined and reduced, written and re-written and constantly compared to the transcribed interviews until a preliminary structure of main themes and sub-themes was identified. The preliminary themes were discussed with colleagues with experience of working with qualitative methods, including hermeneutical phenomenology. In addition, some of these colleagues live with migraine. During the process of analyzing and reflective writing, our understanding evolved and the essence was captured.

### Justification of the study

The quality of phenomenological research could be strengthened by, clarifying how the principles of phenomenological philosophy are implemented in the study [27]. In our study, we adopted van Manen's ([24] p. 12) way of explaining hermeneutic phenomenological research as “a search for the fullness of living,” which was understood as: the different ways a woman with migraine can possibly experience the world as a woman with migraine, and what it means to be a woman living with migraine. According to van Manen [24], phenomenological research starts with curiosity over the meaning of a phenomenon, and researchers need to overcome private feelings and preferences to enable them to strip away scientific conceptions and thereby come to terms with a phenomenon. Therefore, as researchers, we focused on the phenomenon, formulated a phenomenological question driven by a sincere curiosity. Drew [28] emphasize that by keeping the contact with ones own intention to the phenomenon and by keeping a constant reflexion to identify and explain ones unique experience of the phenomenon, validates the study. In this article, we tried to explicate and come to terms with our pre-under-standing by engaging in open and reflective discussions when exploring the women's experience of migraine. Furthermore by discussing the preliminary themes with colleges, we also were challenged about our pre-understandings as our collective understanding of the phenomenon “the meaning of living with migraine” evolved. To ensure that the interpretation we have made is possible and trustworthy, the findings are presented with quotations from the participants [29]

### Ethical considerations

Participation was voluntary and the women were informed that they could change their mind about participating at any time without having to provide an explanation. The women gave their informed consent to their participation, and confidentiality was assured. The Regional Ethical Review Board in Umeå, Umeå University approved the study (Dnr 08-182M).

## Findings

The analysis revealed an essence, three main themes and six sub-themes, which explored the meaning of living with migraine (Table I).

**Table I:** Overview of the essence, main themes, and sub-themes of the phenomenon.

Essence	Being obliged to endure a life accompanied by an unpredictable and invisible disorder
*Main theme*	*Being besieged by an attack*
Sub-theme	Being temporarily incapacitated
Sub-theme	Feeling involuntarily isolated from life
*Main theme*	*Struggling in a life characterized by uncertainty*
Sub-theme	Being in a state of constant readiness
Sub-theme	Worrying about the use of medication
*Main theme*	*Living with an invisible disorder*
Sub-theme	Living with the fear of not being believed
Sub-theme	Struggling to avoid being doubted

### Being besieged by an attack

Having a migraine attack meant being besieged by pain and other symptoms, thereby making it impossible to function as normal. It also meant a temporary lose of both time and of power over life. The analysis revealed two sub-themes: “Being temporarily incapacitated” and “Feeling involuntarily isolated from life."

#### Being temporarily incapacitated

The women could be overwhelmed by a migraine attack that came out of nowhere. An attack influenced the whole body, and produced the feeling that the body was not functioning properly. The headache was incapacitating and was hard to endure, even if it could vary in intensity. Nausea and vomiting enhanced the feeling of being really ill, and sometimes the nausea experienced was worse than the pain itself. The feeling of being incapacitated also derived from visual impairment, communication problems and increased light and noise sensitivity, the latter being described as being magnified to the extent that exposure to light and sound became really painful. This made it impossible to function in certain environments, and could last for hours after the end of a migraine attack.

"The pain is terrible, it's terrible when your are having an attack, it is horrible, you think you will…, well, you just want to fade away so you do not have to feel it, [you would] if it were possible, but you can't."

The incapacity that a migraine attack imposed, led women into more vulnerable situations. Losing the ability to communicate, to see or to function in a normal manner, made women insecure about their capacity to take care of themselves or of others who depended on them, for example, their own child, which sometimes caused fear and anxiety. Women felt that they were recurrently unable to fulfil obligations or perform their best at work, because of migraine attacks and that this put them in a weak position when it came to their opportunities to build a career for themselves or to undertake a career change. Experiencing communication problems, such as not being able to organize thoughts or using the wrong words, as well as not being able to pronounce words because the tongue became numb, was embarrassing and added to the feeling of being vulnerable.

"It [migraine] affects the whole head. It affects memory. If I'm sitting and talking to people, like when I have guests, and I feel that I'm starting to get a migraine, I think “What did they say now?” I do not remember. It is like I can not take it in."

#### Feeling involuntarily isolated from life

Having a migraine attack meant an inability to fully participate in life, while everyone and everything else was carrying on as usual. Women sometimes needed to spend hours in bed, which left them feeling like their life was standing still, and this created anxiety, distress and anger. Even if a migraine attack did not always lead to bed-rest, the women felt abstracted because of their reduced capacity to concentrate and to stay focused on things and people in their immediate surroundings during a migraine attack. When migraine attacks occurred during weekends, it was more burdensome for some women, because weekends normally offered the opportunity to rest and spend time with family and friends.

"You lose your life for a moment… In some ways it is like turning off the water tap and leaving life outside for the moment… to enable you go inside yourself and take care of the headache. It is important for others to know how damned limited and locked up you get."

The involuntary isolation from life extended beyond the attack itself as having to live with uncertainty about being able to fulfil things led to a reluctance to plan ahead and to have dreams for the future, such as going travelling or having a party. As a woman said, “I would never ever plan my own wedding; imagine if I couldn't attend because of a migraine.” Recurrently needing to cancel activities and thereby, possibly, spoil things for others was experienced as being harder than the symptoms themselves, which led to less desire to do things and fewer social contacts.

"The worry about having a migraine attack takes away the joy of planning something joyful. You think “This is going to be fun!” but then you think I might get a migraine. I have probably never felt real joy because of always having this in the back of my mind; it is always present."

### Struggling in a life characterized by uncertainty

As migraine is unpredictable, women were challenged by both trying to increase their own control and by adjusting their lives to the uncertainty, which led to having to live their lives in state of constant readiness. Women experienced relief from using medication, but worried about the long-term health implications and lacked satisfactory alternative treatments. The analysis revealed two sub-themes “Living in a constant state of readiness” and “Worrying about the use of medication."

#### Living in a constant state of readiness

The women never lost hope that the migraine would disappear at some time in the future, and in the meantime they tried to cope with the uncertainty. Learning to live with migraine was a process of adjusting, which meant constantly being on guard and always considering what would happen if they had a migraine before committing themselves to engage in activities. The adjustment process included trying to even out the demands that life made on them and their time and energy by, striving to balance the demands of life.

Living with migraine meant having the feeling of not being in control, and the women struggled to increase their sense of control over their illness. The notion of control did not only involve being able to make plans and realize them, but also restrictions on the activities undertaken and food eaten because of the fear of triggering a migraine. Sometimes the women felt that they were controlled by the migraine, which was described by one woman with a metaphor, “It's as though I am forced to live with somebody who always interrupts and decides what I should or should not do.” Living life in a constant state of readiness meant always searching for and trying to avoid anything that would trigger a migraine to gain control over them. However, the participants’ sensitivity varied, so potential triggers might prove unproblematic on one occasion, but then subsequent exposure might then trigger a migraine, which aggravated the possibility to gain control over the triggers. As stress was a common trigger, women tried to both control it, through exercise, relaxation and mental training and to adjust their lives to avoid stressful situations. Being in a constant state of readiness meant, however, that limitations were inevitably imposed on life owing to the adjustments made. The adjustments included aspects like always needing to sleep at a given time, and making restrictions on what to eat and drink, which sometimes left the women feeling lonely and different from others, as well as sad over how their life had turned out.

"You learn to live with it and you do not know what life would be without it, but it is like permanently wearing a backpack, which is tough, you must always consider the possibility not being able to do things."

#### Worrying about the use of medication

Women had tested different treatments for migraine and experienced different outcomes, but nevertheless they considered prescribed medication to be the key to preventing or stopping their migraine attacks. On most occasions, medication provided relief and made life bearable during a migraine. Nonetheless, using medication was never an easy solution as it raised fears about the negative affect long-term use would have on the body and it was associated with insecurity about the risk of becoming addicted. Furthermore, the use of medication could not restore full function; on the contrary, it was associated with feeling dizzy, tired and having a decreased ability to concentrate. The women's misgivings about taking medication made them hesitate and negotiate with themselves before taking medication, but in the end they often felt that they had no alternative; “… taking medicine all your life! How sort of solution is that? But I do not have a choice because otherwise I would feel so bad I would not be able to live at all.” Having well-functioning medicine available for use increased the feeling of control over migraine and served as a preventative against the most severe migraine symptoms, but it also caused worry, about forgetting to always have the medicine to hand. Thereby medication could be said to ease the burden in some ways, but it added to the consequences of living a life in uncertainty.

"Thanks to the new medication, I can handle it [the migraines] now…. I do not panic about getting migraine, so in that way I have gained a new life, because I had a feeling of panic as migraine is so awful, that you get handicapped by it."

### Living with an invisible disorder

Central to all of the women's narratives was that migraine was an invisible disorder, and that the women were often doubted and had a deep desire to be believed. The women struggled to hide their symptoms and pushed themselves to avoid being doubted. The analysis revealed two sub-themes, “Living with fear of not being believed” and “Struggling to avoid being doubted."

#### Living with the fear of not being believed

The feeling of not being believed was one of the most burdensome aspects of living with migraine and this led to feelings of shame about being afflicted by migraine. The women's experiences of not being understood referred mostly to the invisibility of migraine and the fact that no one else can see or experience how painful and dreadful it is. The women narrated that migraine was perceived as being the same as a headache by society and, therefore, that many people interpreted a migraine as being an insignificant problem. This meant that migraine was not perceived to be an acceptable excuse for not being able to participate in social commitments or perform duties. The sense of not being believed made the women feel that their willingness to do things and to participate in social activities was being brought into question, and because of this, they feared being perceived as either a weak or a lazy person. Moreover, the women worried that their colleagues would think that they were feigning illness if they had to be on sick leave for a day.

"People can believe that it [migraine] is an excuse for not going somewhere, it is stupid not to say [that you have migraine], but it is like you feel ashamed sometimes… you do not want people to think you are a wimp."

A sense of not being believed could also occur when meeting professionals in the healthcare services. Occasionally, they were met with negligence, which led to the feeling that they were not being taken seriously. However, when feeling that their problem was being acknowledged, a sense of security occurred among the women. When the women encountered staff in the health service without a great deal of knowledge of migraine, their lack of understanding contributed to feeling that it was not worth searching healthcare other than to renew prescriptions.

#### Struggling to avoid being doubted

To avoid being doubted the women pushed themselves to continue working despite having a migraine attack. They also tried to keep ahead of their workload in case they became sick later on during the week. During severe attacks, the women had no choice but to stay at home and, between attacks, they tried to live a normal healthy life, undertaking the same workload as any person without migraine. The threat of being doubted made the women unwilling to tell those in their surroundings about their migraine, and they tried to hide the symptoms. This meant that the perpetual worry, the limitations inflicted by migraine on their life to avoid migraine also remained invisible to others. In contrast, however, when others revealed their confidence, it resulted in a feeling that there was less pressure to hide symptoms and it enabled the participant to take the time to recover, which in turn led to less migraine attacks.

"Two years ago I ended up in the hospital. It was impossible to go on. Hopefully one learns from [an experience like] this. I believe that with increased confidence from [colleagues at] work, one dares to stay at home and rest. At the beginning I just went on… and then the migraine got ten times worse."

### Essence: Being obliged to endure a life accompanied by an unpredictable and invisible disorder

Migraine permanently accompanied the women through life, whether by means of its presence or in the form of a perpetual threat that needed to be taken into account. It was characterized by its invisibility and its unpredictability. Migraine could strike hard without any notice, and was incapacitating to the extent that the woman had to slow down her life, or even put it on hold for the moment. The women were obliged to make adjustments and fought a never-ending struggle to control and avoid their migraine, as well as to endure the attacks. The women also struggled to maintain their activities when inflicted by a migraine, partly out of fear of losing the confidence of others who might not believe that migraine was a genuinely debilitating illness.

## Discussion

This study was intended to explore the meaning of living with migraine. The essential meaning of living with migraine was understood as “Being obliged to endure a life accompanied by an unpredictable and invisible disorder.” This indicates that living with migraine means having to endure life with a disorder that is not a constant source of pain, but which directs and rules one's life, owing to the perpetual threat of that one will become incapacitated. The invisibility of the disorder and the feeling of being doubted about whether one was really being afflicted with migraine further complicates life. The knowledge provided by the research presented here adds a new perspective revealing the deep meaning of living with migraine, and it can be seen as a complement to existing research, which has pointed out the impact migraine has on life [20-22] as well as on the strategies adopted to manage it [16-19].

The women struggled with a life of uncertainty, not knowing when the attacks would strike and lived with fear of not being able to take care of themselves or of their dependents during an attack. The unpredictability of migraine resulted in the need for the women to anticipate and be prepared for an eventual attack of migraine, as a result of which, they could never let their guard down. This could be understood to be a strategy used by the women to cope with their migraine, and it mirrors the findings of Meyer [19], who described vigilance in women with migraine as “the art of watching out,” and explained that women scanned the environment, both internally and externally for changes or possible indications of change.

In this research, the meaning of having a migraine attack was understood as being besieged by an attack. This differentiates our study from earlier ones that offered descriptions of the symptoms [17,20,22]. The meaning of being besieged arose because the body was temporarily dominated by the symptoms of the migraine attack, which made it impossible to function and fully participate in life and forced the women to put aside everything else to deal with their migraine. Medication could alleviate the symptoms, but the women did not fully recover until the attack was over, and, on an ongoing basis, they still were preoccupied with the potential threat of migraine and with the measures required to avoid it.

The use of medication both eased and compounded the consequences of living in a state of uncertainty. It contributed to the feeling of having some control over life, but it also caused worries about the long-term consequences of taking medication and the risk of becoming addicted. This made women negotiate with themselves before taking medicine. This could be understood in the light of Meyer's [19] and Peters et al. [18] research, where people with migraine calculated the risk of taking medication to determine whether the benefits of the treatment outweighed the consequences. The women in our study felt that they did not actually have a choice other than to take medication, something that the women in Meyer’s study [19] did not express explicitly, however when it came to situations when they needed to be able to function, they took migraine medicine as first choice.

Living with migraine meant having to live with an invisible disorder, which influenced the women's experience of not being believed. This is in line with research conducted by Soderberg et al. [30], who described how invisible symptoms associated with fibromyalgia also led to the feeling of not being believed by others. In our study, the feeling of being doubted came to the fore when those in the woman's immediate surroundings viewed migraine as an insignificant problem, more like an ordinary headache. The women stated that the invisibility of pain was the main reason for them not being believed. Holloway [31] described how the very invisibility of pain caused persons with chronic lower back pain problems of being discredited. Some symptoms of migraine may, however, have the reverse effect, such as that described by Moloney et al. [22], who said that the visibility of vomiting as a symptom validated the seriousness of the headache to others.

It might seem paradoxical that the women in this study wished for more understanding at the same time as they tried to keep others from knowing about their disease, however, this could be understood as a struggle between the benefits of being understood and the fear of not being believed. The importance of being understood emerges in Olsson's et al. [32] research, where women with multiple sclerosis revealed that feeling understood was central to feeling well despite their illness. Furthermore, the participants in an investigation by Lun et al. [33] reported higher life satisfaction and fewer physical symptoms on days when they felt understood by others. When the women in our study felt trusted, they did not need to hide their symptoms and could take the time required to recover, which led to a decrease in the number of migraine attacks. However, to our knowledge, this has not been shown in migraine research previously and more research is needed about the importance of being understood when living with migraine.

Experience of being doubted was, however, a burdensome aspect of living with migraine and this was in line with Cottrell et al. [21], where misunderstanding by others was highlighted as being an area people with migraine found most difficult to live with. The women in our study experienced guilt about having migraine as a result of others’ perceptions that migraine was an excuse to avoid responsibilities, and Moloney et al. [22] referred to women's feeling of guilt because of the stigma that migraine could be avoided if the women exerted sufficient self-discipline. The women in our study struggled to fulfil their obligations and not cancel things when they had a migraine attack to avoid being doubted. This could be understood as an avoidance of stigmatization in accordance with Holloway [31], who described how avoidance of stigmatization may lead either to risk behaviours that might exacerbate pain, or to concealment and social isolation. Owing to the uncertainty of having a migraine attack, the women in our study sometimes refused invitations and social engagements, which could lead to social isolation, a finding that emerged in a study by Ruiz de Velasco et al. [20], where persons with migraine lost contacts with their friends and reported varying degrees of social isolation. It is possible that hiding migraine symptoms, as the women stated they did, might lead to them not seeking social support, which was apparent in the research of Gunel and Akkaya [34], where people with migraine used social support less than a group without migraine. In addition to not seeking help, Moloney et al. [22] argued that the existing stigma of migraine decreases the likelihood of a person with migraine being able to obtain appropriate treatment and social support.

Women in this study lived with the fear of not being believed and, when meeting healthcare professionals, they had experienced both that their illness was acknowledged and that they were not being taken seriously. According to Ramsey [35], some relief can be provided when women with migraine are recognized as truly suffering. In addition, Buse et al. [6] argued that the care of persons with migraine can improve when healthcare providers have an increased understanding of and communicate about the burden of migraine. Our study increases knowledge of the meaning of living with migraine and improves the understanding of what life with migraine can be like.

### Methodological considerations

This research relied on the narrative accounts of ten women and on their reflections to provide an understanding of the phenomenon from the inside, from the participants’ perspective [24]. One limitation might be that it was only women who were represented, and therefore we do not know if the findings would have been different if men were to have participated. Another limitation is that all of the women were members of the Swedish Migraine Association, and therefore it is possible that they were more active in handling their migraine than the average person with migraine. However, they all stated that the only engagement they had in the association was reading the magazine.

The collected data contained rich information and was, therefore, judged to be adequate to meet the aims of this study. According to Norlyk and Harder [27], the sampling criteria in phenomenological research should be people with experience of the phenomenon under consideration and that the variation within the phenomenological framework reflects variation in the experiences of the participants. The interviews were about two and a half hours long, which indicates the richness of the experience of living with migraine. The drawing of the picture was performed when the woman felt that she had no more to add to her narrative, which mostly occurred one to one and a half hours into the interview. The women were promised that, if they were prepared to attempt to make a drawing of what it is like having to live with migraine, then their pictures would be neither reproduced, nor displayed, without their permission. This resulted in all of the women but two drawing an image. The use of the pictures enriched the discussions and we can report that valuable additional information was obtained directly through their use. This is in accordance with the findings of Guillemin [36], who concluded that drawing pictures is a process of knowledge production about the illness itself and that it offers further insight into how people understand their illness. Guillemin [36] also argued that when the participants make their own interpretation of their drawing, it further validates the drawing as a research method and it can be used together with other methods of data collection, for example interviews. After concluding the analysis and the writing process, we asked one of the women for her consent to reproduce her drawing in the article. This drawing is to be seen as an example, as all the drawings were different from each other, as they reflect each woman's unique experiences.

## Conclusions

The findings show that living with migraine means living life in a state of uncertainty, being engaged in a constant struggle to maintain control, and living with the fear of not being believed. Having a migraine attack is more than having a headache; it means that one is temporarily besieged by pain and other symptoms, which makes it impossible to function normally. The treatment of migraine must involve more than just treating the symptoms; thus, it is important to explore and acknowledge what it means to live with migraine, to ensure that those afflicted will encounter greater understanding.
